# Investigating the role of glycoprotein hormone GPA2/GPB5 signaling in reproduction in adult female *Rhodnius prolixus*

**DOI:** 10.3389/finsc.2022.1096089

**Published:** 2022-12-22

**Authors:** Areej N. Al-Dailami, Ian Orchard, Angela B. Lange

**Affiliations:** Department of Biology, University of Toronto Mississauga, Mississauga, ON, Canada

**Keywords:** triatomine, kissing bug, reproduction, egg production, immunohistochemistry, qPCR, transcript

## Abstract

Glycoprotein hormones are essential for regulating various physiological activities in vertebrates and invertebrates. In vertebrates, the classical glycoprotein hormones include follicle-stimulating hormone (FSH), luteinizing hormone (LH), thyroid-stimulating hormone (TSH) and chorionic gonadotropin (CG), which have crucial roles in growth, development, metabolism, and reproduction. In female mammals, FSH stimulates egg production in the ovaries, whereas LH and CG act as the triggers for follicular ovulation. The more recently discovered heterodimeric glycoprotein hormone GPA2/GPB5 (called thyrostimulin in vertebrates) is suggested to be involved in reproductive processes in arthropods. Here, we focus on understanding the role of GPA2/GPB5 and its receptor, LGR1, in the reproductive success of adult female *Rhodnius prolixus*, a vector of Chagas disease. qPCR was used to monitor the expression of *GPA2* and *GPB5* transcripts and their receptor in different tissues. Immunohistochemistry was used to show the distribution of *GPB5* in the nervous system and reproductive system, and RNA interference was used to disrupt the glycoprotein hormone signaling pathway. Both subunit transcripts, *GPA2* and *GPB5*, are present in a variety of tissues, with the greatest expression in the central nervous system; whereas the *LGR1* transcript is present in peripheral tissues, including the fat body and the reproductive system of adult females. In the adult female, GPB5-like immunoreactive axonal projections are present in the trunk nerves extending onto the reproductive tissues, with processes overlaying the ovaries, oviducts, spermatheca, and bursa, indicating the possibility of neural control by neurons containing GPA2/GPB5. In addition, GPB5-like immunostaining is present in muscles encircling the ovarioles, and in the cytoplasm of trophocytes (nurse cells) located in the tropharium. GPB5-like immunoreactive processes and blebs are also localized to the previtellogenic follicles, suggesting an involvement of this glycoprotein hormone signaling in oocyte development. *LGR1* transcript expression increases in the adult female reproductive system post-feeding, a stimulus that initiates reproductive development, adding further support to an involvement in reproduction. We have investigated the effect of *LGR1* downregulation on reproductive processes, monitoring the number and the quality of eggs laid, hatching ratio, and production of vitellogenin (Vg), the major yolk protein for developing eggs. Downregulation of *LGR1* leads to increases in transcript expression of vitellogenin, *RhoprVg1*, in the fat body and the vitellogenin receptor, *RhoprVgR*, in the ovaries. Total protein in the fat body and hemolymph of dsLGR1-injected insects increased compared to controls and associated with this effect was a significant increase in vitellogenin in these tissues. dsLGR1-injection leads to accelerated oogenesis, an increase in the number of eggs produced and laid, an increase in egg size and a reduction in hatching rate. Our results indicate that GPA2/GPB5 signaling acts to delay egg production in adult female *R. prolixus.*

## 1 Introduction

Glycoprotein hormones (GPHs) are important in controlling several physiological processes and behaviors in vertebrates, including reproduction, energy metabolism, growth, and development (1). The GPH family includes follicle stimulating hormone (FSH), luteinizing hormone (LH), thyroid-stimulating hormone (TSH) and chorionic gonadotropin (CG) (1–4). Each GPH is formed by the heterodimerization of a common alpha subunit (GPA1) and a hormone specific beta subunit, which confers the binding site specificity for the receptor (1, 5). The latest GPH discovered, called thyrostimulin, is, however, comprised of a distinct alpha subunit (GPA2) and its hormone specific subunit GPB5. Unlike the other vertebrate GPHs that are restricted to vertebrates, genes for GPA2 and GPB5 are found in most bilaterians (6–10). Glycoprotein hormones bind to receptors that belong to a unique superfamily of G protein-coupled receptors (GPCRs) called the leucine-rich repeat-containing GPCRs (LGR). Type A LGR (Rhodopsin type) includes the receptors for TSH, FSH, LH and CG, as well as for GPA2/GPB5. In invertebrates, GPA2/GPB5 binds to a receptor named LGR1, which shares more than 50% amino acid sequence homology to the membrane-spanning regions of the classic GPH receptors (6, 11–14).

There is limited information regarding the biological functions of thyrostimulin in vertebrates. It is believed that thyrostimulin is involved in several physiological processes such as stimulating thyroxine production (9, 15), osteoblastic bone formation (16), and that it can act as a paracrine factor locally to regulate reproduction in the mammalian ovary (17). In invertebrates, GPA2/GPB5 has only been studied in small number of species but it appears to play potential roles in development (13, 18–20), in salt and water balance (13, 14, 19–21), and in reproduction (22, 23).

In the fruit fly Drosophila melanogaster, *the* GPA2/GPB5 receptor transcript (*DLGR1*) levels increase during life-transitioning stages with a very prominent peak in third instar larvae, the last stage before pupal formation, followed by a decline in early pupal stages (6, 19). Silencing *DLGR1* significantly disrupts metamorphosis leading to developmental defects, including suppression of white pupa formation in D. melanogaster larvae that is associated with reduced ecdysteroid titer (19). In addition, *DLGR1* transcripts are highly expressed in the hindgut, Malpighian tubules, and other tissues with water transporting epithelia (21). *DLGR1* knockdown flies are less tolerant to dehydration stress (19), indicating that GPA2/GPB5 is likely involved in osmoregulation in addition to its proposed developmental role. Also in the mosquito, Aedes aegypti, GPA2/GPB5 is involved in regulating hydromineral balance and appears to maintain ionic homeostasis through inhibiting natriuresis and promoting kaliuresis (13). In addition, though, GPA2/GPB5 appears to play a role in spermatogenesis and silencing *LGR1* in adult male A. aegypti results in shorter sperm flagellar lengths, a significant reduction of spermatozoa in testes, and when these males are mated with females, a significant reduction in the number of eggs hatching (22). Another role in reproduction is found in the female prawn, *Macrobrachium rosenbergii*, where downregulation of *GPA2/GPB5* transcripts results in a reduction of vitellogenin transcript expression levels (*MrVg*) in the hepatopancreas, and knockdown of *MrLGR1* increases *MrVg* receptor (*MrVgR*) transcript expression in the ovaries. These result in larger oocytes, suggesting GPA2/GPB5 acts as a gonad-inhibiting factor in the eyestalk-hepatopancreas-ovary endocrine axis in *M. rosenbergii* (23).

*Rhodnius prolixus* is a blood-feeding hemipteran, and serves as a principal vector of *Trypansoma cruzi*, the parasite causing Chagas disease (24, 25). In a recent report on the glycoprotein hormone GPA2/GPB5 in *R. prolixus*, the GPA2 and GPB5 subunits, and LGR1, were cloned and shown to have the unique structural features shared by GPH subunits, including the ten conserved cysteine residues of critical importance for disulfide bridge formation and important for cystine-knot formation; a main characteristic of this GPH subfamily (14). There is one predicted N-glycosylation site on GPB5 at Asn160, and it is suggested that glycosylation sites are important for dimer formation. In *D*. melanogaster *and A. aegypti*, GPA2/GPB5 heterodimers activate LGR1 transfected into HEK293 cells, supporting the heterodimerization phenomenon (12, 26). However, the difference in expression profiles of GPA2 and GPB5 reported in several studies suggest the possibility of the subunits functioning as homodimers (8, 17). Whether the GPA2/GPB5 glycoprotein hormones function as heterodimers, homodimers or both, remains to be elucidated.

Interestingly, we recently found that GPA2/GPB5 signaling in *R. prolixus* is involved in promoting blood-gorging and appears to assist in tolerating prolonged unfed conditions that *R. prolixus* often experience (14). Blood-gorging is also the stimulus for egg production in *R. prolixus* and interestingly, a transcriptomic analysis of the main tissues involved in egg production in *R. prolixus* suggests that the GPA2/GPB5 signaling pathway might be involved in regulating reproductive physiology (27). Numerous studies have examined the control over egg production in adult female *R. prolixus*, and these have revealed complex neural and hormonal elements allowing for a tightly synchronized and integrated behavior (see 27). The aim of this study, therefore, is to explore the role of GPA2/GPB5 signaling in adult female *R. prolixus* egg production, using physiological and molecular techniques, including RNA interference.

## 2 Materials and methods

### 2.1 Animals

Experiments were performed on adult *R. prolixus* females taken from an established colony at the University of Toronto Mississauga, maintained in an incubator in the dark at 25°C and 50% humidity following the method reported by Orchard etal. (28). For the dsRNA experiments, insects were housed in an incubator maintained at 28°C on a 12:12 h light/dark cycle a week prior to dsRNA injections and throughout the experiment. All insects used in this work had a similar feeding and body weight history.

### 2.2 Whole mount immunohistochemistry

Central nervous systems (CNSs) and reproductive tissues of unfed, unmated adult female insects were dissected in *R, prolixus* physiological saline (150 mM NaCl, 8.6 mM KCl, 2 mM CaCl_2_, 4 mM NaHCO_3_, 34 mM glucose, 8.5 mM MgCl_2_, 5 mM HEPES, pH 7) at room temperature. Fixation and staining were performed as described by Al-Dailami etal. (14), using a primary anti-GPB5 antiserum raised in rabbit at a concentration of 1:1000 in phosphate buffered saline (PBS, 6.6 mM Na_2_HPO_4_/KH_2_PO_4_, 150 mM NaCl, pH 7.4) with 0.4% Triton-X/2% normal goat serum (NGS)/2% bovine serum albumin (BSA). The anit-GPB5 antiserum was custom made against a conserved sequence within GPB5 (CDSNEISDWRFP) by Rocco and Paluzzi (26) and kindly provided by Prof. J.P. Paluzzi, York University, Canada. Tissues were incubated for 48 h at 4°C. Following washes in PBS, the tissues were incubated for 24 h in secondary antibody (1:600 Alexa Fluor 488-conjugated goat anti-rabbit Ab (Life Technologies, Carlsbad, CA), made up in PBS containing 10% NGS). Tissues were mounted on cover slips with one drop of Fluoroshield (Sigma-Aldrich, ON, Canada). Controls were prepared following the same protocol but with preabsorption of the antiserum with 10^−5^ M antigen (CDSNEISDWRFP) overnight at 4 °C prior to use.

Images were acquired using Z-stacks obtained by a confocal microscope LSM-800 (Carl Zeiss, Jena, Germany) and then processed with the Zeiss LSM Image Browser software. Z-stacks were prepared using ImageJ Software (https://imagej.nih.gov/ij/).

### 2.3 RNA extraction and reverse transcription quantitative PCR

Total RNA was extracted from tissues of adult female *R. prolixus* using TRIzol reagent (Invitrogen by Thermo Fisher Scientific, MA, USA) according to the manufacturer's instructions. cDNA samples were synthesized using the High-Capacity cDNA Reverse Transcription Kit (Applied Biosystems, Mississauga, ON, Canada), using 1 µg of total RNA, random primers and 50 U of MultiScribe MuLV reverse transcriptase. qPCR assays were performed as previously described (14). Actin and Rp49 (60S ribosomal protein) housekeeping genes were used as reference genes to normalize the target gene expression. To assess the accuracy of cDNA product amplification, the dissociation curves were examined and found to have a single peak produced for each pair of primers (**Supplementary Table 1**). Transcript abundance was normalized to transcript levels of the housekeeping genes following the 2^-ΔΔCt^ method (29). All samples had 5 - 6 biological replicates with each containing 2 technical replicates and using no-template controls.

### 2.4 Double-stranded RNA synthesis

To synthesize double-stranded RNA (dsRNA), two non-overlapping fragments of *LGR1* were prepared by PCRs by conjugating the T7 RNA polymerase promoter (5′-taatacgactcactatagggaga-3′) to the 5′ end of the gene specific primers (**Supplementary Table 1**). dsRNA synthesis was performed as described previously in Al-Dailami etal. (14).

### 2.5 dsRNA delivery

Seventeen adult female insects were each injected with 3 µL of dsLGR1 (containing 2 µg of two non-overlapping dsRNA) into the thoracic/abdominal hemocoel at the base of the metathoracic legs using a 10 μL Hamilton micro syringe (Hamilton Company, NV, USA). A second group consisting of 21 insects were injected with 3 µL containing 2 µg of ds ampicillin resistance gene (dsARG) as control. All insects were left for 1 h at room temperature and then placed into an incubator at 28°C with a 12:12 light/dark cycle. Knockdown of the *LGR1* transcript was measured in the ovaries and fat body at 2d, 4d and 14d following injection using qPCR (**Supplementary Figure 1**).

### 2.6 Feeding and egg production assays

Virgin adult females (10 days post ecdysis) were given access to a blood meal and those that fed properly (~3 times their body weight) were used in the experiments. After feeding, each female insect was then kept in a cubicle containing two fed adult males and allowed to copulate for two days and then injected with dsRNA, as described above. At 6 days post blood meal (PBM), the hemolymph, fat body, and ovaries (minus chorionated eggs) were collected and processed for RT-qPCR, ELISA, Western blot, or protein determination. Insects from each group were used to examine ovarian morphology and monitored for egg laying (photographed with a Leica DVM6 digital microscope (Leica Microsystems, Wetzlar, Germany), and hatchability. Measurements of length and width of eggs were recorded using ImageJ software to determine differences between treatment groups, and the volume was determined using the following equation of an ellipsoid = $\frac{\text{π}}{6}\;{\left(Width\right)}^{2}\left(length\right)$ given the circular nature of the longitudinal axis (30).

### 2.7 Sample collection and total protein quantification

Hemolymph and fat body samples were collected from dsARG-injected and dsLGR-injected adult female insects at 6 days PBM for total protein and vitellogenin measurements. Insects were immobilized on surgical wax and 10 uL of hemolymph collected using a Hamilton syringe (Hamilton Company, Reno, NV, United States) from cut legs while gently pressing the abdomen. The hemolymph was placed in ice-cold microtubes and then diluted in cold anticoagulant solution (10 mM Na2-EDTA, 100 mM glucose, 62 mM NaCl, 30 mM sodium citrate, 26 mM citric acid, pH 4.6) at a ratio of 1:5 (anticoagulant: hemolymph) (31). Samples were then centrifuged at 10,000 × g for 10 min at 4°C to remove hemocytes and the supernatants used for determination of vitellogenin concentration.

After hemolymph collection, fat bodies were carefully dissected under cold *R. prolixus* saline and protein extraction performed with TRIzol reagent (Invitrogen by Thermo Fisher Scientific, MA, USA) according to the manufacturer’s recommendations. Following protein extraction, quantification of total protein was performed using the BCA protein quantification assay (Pierce™ BCA Protein Assay Kit, Thermo Fisher, ON, Canada).

### 2.8 Vitellogenin quantification by ELISA

Vitellogenin quantification in the hemolymph and fat bodies was carried out using an enzyme-linked immunosorbent assay (ELISA) as described by Aguirre etal. (32) and Leyria etal. (33). Microtiter plates were loaded with 100 μL/well of vitellogenin as standard or with the appropriate dilutions of hemolymph and fat body in carbonate buffer (15 mM Na_2_CO_3_, 35 mM NaHCO_3_, pH 9.6) and incubated for 90 min at 37 °C. Following incubation, plates were washed two times with phosphate buffered saline -Tween (PBST: 8.2 mM Na_2_HPO_4_, 1.5 mM KH_2_PO_4_, 150 mM NaCl, 2.7 mM KCl, 0.05% Tween 20, pH 7.4) on a shaker at room temperature for 5 minutes for each wash. Plates were loaded with 100 µL of affinity purified rabbit IgG Vg antibody at a concentration of (0.01 µg/mL) prepared in PBST containing 0.1% BSA for 60 minutes at 37°C. The polyclonal anti-Vg antibody was designed commercially by Boster Biological Technology (CA, USA), using a fragment of the RhoprVg sequence (PLPQFVLQSRPELVPLPKLVAGGQVLDIVKTKNYSNCEQRMAYHFGLTGLTDWEPASNQ) and found to be specific (33). Plates were then washed two times as described above followed by loading plates with 100 µL of solution containing anti-rabbit immunoglobulin conjugated to horseradish peroxidase (HPR) in PBST (1:4000) for 30 minutes at 37°C. After washing, plates were incubated with 100 µL of the enzyme 3,3′,5,5′-Tetramethylbenzidine (TMB) Liquid Substrate System (Millipore-Sigma, Oakville, ON, Canada) for 15 minutes and then the reaction was stopped by adding 100µL of 1M H_2_SO_4_. Plates were read at 410 nm using a multi-mode reader (Synergy HTX, CA USA).

### 2.9 SDS-PAGE and Western blot

For SDS-PAGE, pre-made gels (percentage 4 – 20 %, Mini-Protean TGX Stain-Free Precast Gels, BioRad, ON, Canada) were used to separate proteins from 1 μL of hemolymph under reducing conditions, following the manufacturer's instructions. After electrophoresis, the gel was stained with QC Colloidal Coomassie (BioRad), for 1 h at room temperature with gentle shaking. The gel was then destained over night at 4^o^C with gentle agitation to remove the background using a destaining solution of 50 % methanol in water with 10% acetic acid. The gel was imaged on a ChemiDoc XRS system (BioRad). For western blot, 1 μL of hemolymph (1:20 dilution) was used to separate proteins under reducing conditions on pre-made gels, following the method presented by Leyria etal. (31). The gels were incubated in anti-vitellogenin antibody at a 1:2000 dilution overnight at 4°C with gentle agitation. For the secondary antibody, HPR-conjugated goat anti-rabbit IgG was used at a dilution of 1:5000 and the gel was incubated for 1 h at room temperature with gentle shaking. Blots were visualized using enhanced chemiluminescence (Clarity Western ECL Substrate, BioRad), imaged on a ChemiDoc XRS system and analyzed using Image Lab 5.0 (BioRad Software and System).

### 2.10 Statistical analyses

All graphs were created using the GraphPad Prism Software (GraphPad Software, CA, USA). Significance of differences were determined either with Student's t-test or one-way ANOVA followed by Tukey’s test as indicated. The average cumulative eggs laid per female per day was analyzed by fitting the data with regression lines and comparing the slopes for significant differences using an F-test.

## 3 Results

### 3.1 GPB5-like immunoreactivity associated with trunk nerves and female reproductive tissues

**Figure 1:**
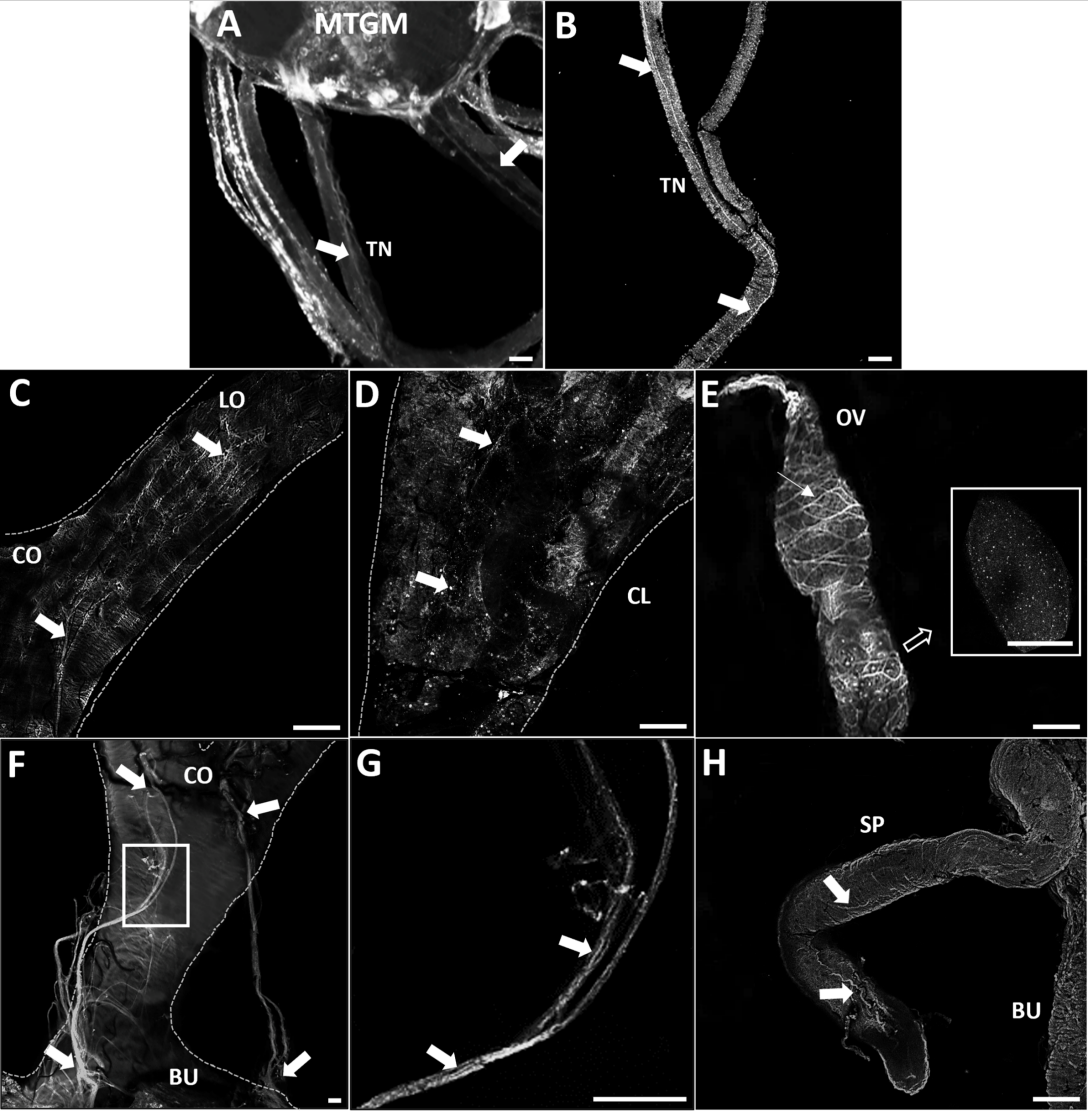
GPB5-like immunoreactivity associated with the trunk nerves and reproductive tissues of adult female *R. prolixus*. **(A)** Mesothoracic ganglionic mass (MTGM) with attached abdominal nerves displaying GPB5-like immunoreactive processes in the trunk nerves (TN) (arrows). **(B)** GPB5-like immunoreactive processes projecting from the trunk nerve to the reproductive tissues (arrows). **(C)** Common oviduct (CO) and lateral oviduct (LO) containing GPB5-like immunoreactive processes (arrows) extending along the LO to the calyx (CL) shown in **(D)** (arrows). **(E)** GPB5-like immunoreactive processes and blebs are present in the previtellogenic follicles of the ovarioles (inset). GPB5-like staining is also found in muscle fibers encircling the ovariole forming a criss-cross pattern (thin arrow). **(F)** GPB5-like immunoreactive axons are present in nerves that project to the bursa (BU), as indicated by arrows. **(G)** Enlargement of boxed area in F showing GPB5-like immunoreactive processes in the nerves (arrows). **(H)** GPB5-like immunoreactive processes in the spermatheca (SP) (arrows). These are representative images obtained from 10 preparations. Scale bars: **(A, B)** are 50 μm, **(C–E)** and **(H)** are 100 μm, **(F–G)** 20 μm.

**Figure 2:**
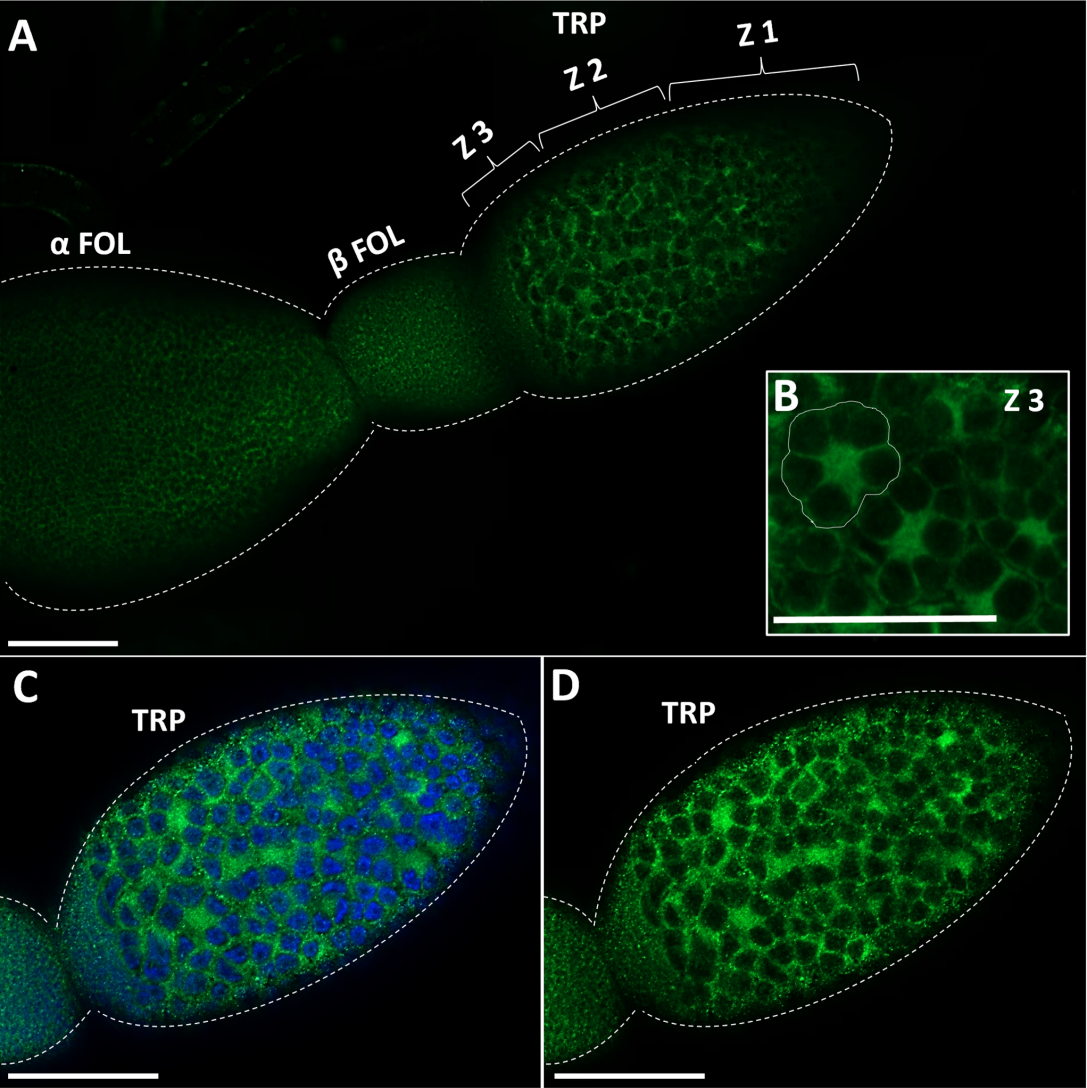
Immunostaining of GPB5-like staining in the ovariole in adult female *R. prolixus.* **(A)** GPB5-like staining (in green) is observed in the processes over the follicle cells as well as in the cytoplasm of trophocytes (nurse cells). GPB5-like staining is brightest in Zone 3 of the tropharium where nuclear aggregates are peripherally arranged around the central core of the cytoplasm (encircled in white) **(B–D)** DAPI staining (in blue) shows GPB5-like staining in the cytoplasm around the large nuclei in the tropharium. TRP, tropharium; α FOL, alpha follicle (terminal follicle); β FOL, beta follicle (terminal follicle); Z1, zone 1; Z2, zone 2; Z3, Zone 3. Similar results were obtained in 10 preparations. Scale bars: **(A–D)** 100 μm.

GPB5-like immunoreactivity is found in cells and processes throughout the adult female CNS, as previously reported for fifth instars (14). Immunoreactive axons are also found in the median trunk nerves (**Figures 1A, B**, arrows) which project to the reproductive tissues of adult female *R. prolixus* (for anatomy of the reproductive system see 27). GPB5-like immunoreactive processes are present along the lateral oviducts (**Figure 1C**, arrows), extending from the junction of the trunk nerve and down the common oviduct (**Figures 1F, G**). Processes and blebs are found distributed on the calyx (**Figure 1D**, arrows) and localized to the tropharium and previtellogenic follicles of the ovarioles (**Figure 1E**). GPB5-like staining of muscle fibers encircling the ovariole is also evident (**Figure 1E**, arrow), revealing a criss-cross pattern similar to the phalloidin staining of the muscular sheath as reported by Sedra and Lange (34). GPB5-like immunoreactive processes are also seen on the spermatheca and bursa (**Figure 1H**, arrows), but not on the cement gland. Interestingly, robust GPB5-like immunoreactivity is observed intracellularly within the trophocyte cells of the tropharium (**Figure 2**). The tropharium contains the nurse cells or trophocytes in different stages of development (divided into 3 zones, z1, z2, z3). GPB5-like staining is more evident in the proximal tropharium, mainly in zones 2 and 3 (at these developmental stages cells are organized to form a syncytium in which the nuclei share a common cytoplasm) (**Figure 2B**). DAPI staining of the nuclei is shown in blue merged with GPB5-like staining in green (**Figure 2C, D**). Control samples with preabsorbed antiserum resulted in an absent or greatly reduced GPB5-like staining intensity (**Supplementary Figure 2**).

### 3.2 Tissue distribution of *GPA2*, *GPB5*, and *LGR1* transcripts in adult females

**Figure 3:**
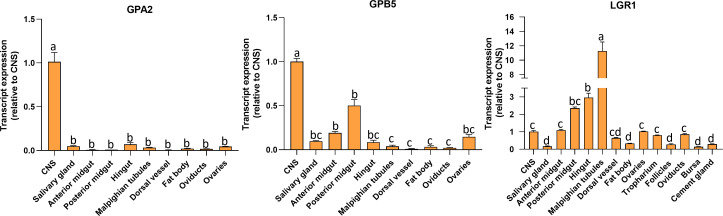
Tissue distribution of *GPA2*, *GPB5*, and *LGR1* transcripts in unfed adult female *Rhodnius prolixus* at 10-12 d post ecdysis. The transcript levels in each tissue were quantified relative to the expression in the CNS (value ~ 1) using qPCR and the 2^-ΔΔCt^ method. The y axes represent the relative expression obtained *via* geometric averaging using *Rp49* and *actin* as reference genes. The results are shown as the mean ± SEM (n = 5-6, where each n represents a pool of tissues from 3 insects). Statistical analysis was performed using a one-way ANOVA and Tukey’s test for *post-hoc* analysis. Significance of P< 0.05 is denoted using letters to indicate bars that are significantly different from others. CNS, central nervous system.

Transcript expression of *GPA2*, *GPB5*, and *LGR1* (**Figure 3**) was investigated in unfed adult females by RT-qPCR. *GPA2* and *GPB5* transcripts are present in a wide range of tissues, with highest expression in the CNS. The posterior midgut exhibited high *GPB5* expression levels followed by the anterior midgut, ovaries, and salivary glands. Interestingly, GPA2 and GPB5 expression profiles are not identical within the tissues, which might suggest the subunits have the potential to form homodimers. *R. prolixus LGR1* transcript expression levels are distributed throughout all tissues examined and found to be highest in the Malpighian tubules, hindgut, and midgut, as previously reported for fifth instars (14). *LGR1* expression is also found in adult female reproductive tissues, including the ovaries and associated structures.

### 3.3 Temporal transcript expression of *LGR1* in the reproductive system of adult females

**Figure 4:**
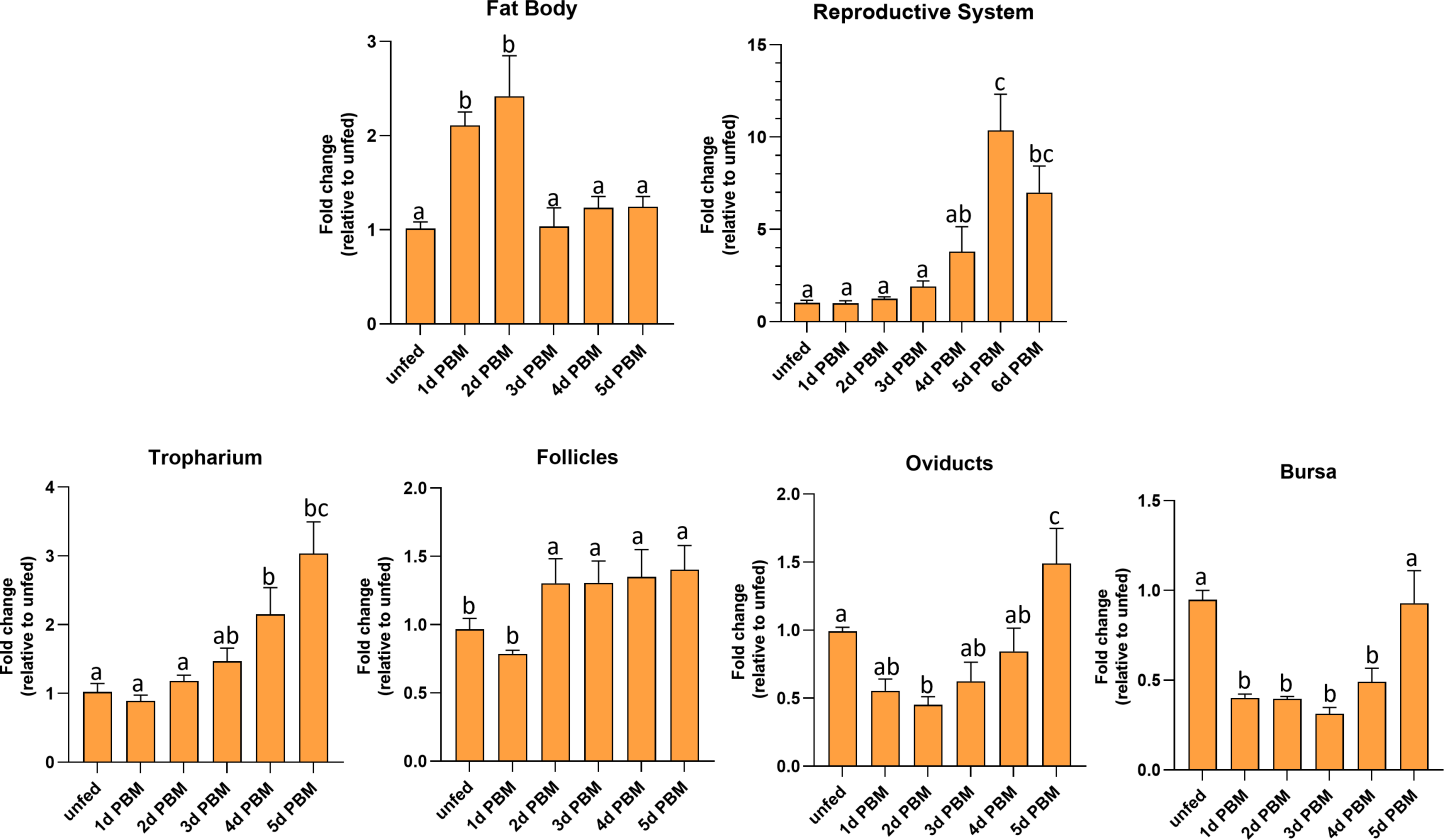
Temporal expression levels of *LGR1* in the fat body and reproductive tissues of adult female R. *prolixus* at 10-12 d post ecdysis. Fat body, reproductive tissues (tropharium, follicles, oviducts, and bursa) were dissected and analyzed for *LGR1* during different nutritional states (unfed and fed) and different time points (unfed; 1 to 6 days post blood meal (PBM). Expression levels of *LGR1* were quantified using RT-qPCR and the 2^− ΔΔCt^ method. Results are shown as mean ± SEM (n = 4-5, where each n represents a pool of tissues from 2 insects). Statistical analysis was performed using a one-way ANOVA test with Tukey's multiple comparisons. Significance of P < 0.05 is denoted using different letters to indicate bars that are significantly different from others.

*R. prolixus LGR1* transcript levels were examined in the FB and reproductive tissues in both unfed and fed adult females (**Figure 4**). The FB (the main producer of yolk precursor proteins (YPPs), including vitellogenin, needed for egg growth) has significantly higher *LGR1* expression levels at 1 and 2 days PBM and a significant decrease at 3 to 5 days PBM returning levels to that seen in unfed insects. *LGR1* in the reproductive system (RS) displayed a gradual increase over 1 to 4 days PBM with a peak at 5 days PBM. *LGR1* transcript levels displayed a similar trend in the various regions of the RS, including the tropharium, follicles, oviducts, and bursa. Taken together, the temporal distribution results indicate that *LGR1* transcript levels increase in FB and RS as the days post-feeding advance and eggs are developing.

### 3.4 Knockdown of *LGR1* transcript and effects on egg production

To investigate the role of GPA2/GPB5 signaling in the reproductive physiology of *R. prolixus* adult females, *LGR1* was downregulated using RNA interference (RNAi). Changes in *LGR1* expression levels were verified in the FB and RS using RT-qPCR, with a reduction of 45% observed at 2 days following dsLGR1-injection relative to the dsARG-injected insects and the level remaining reduced for the duration of the experiment (16 days PBM) (**Supplementary Figure 1**).

**Figure 5:**
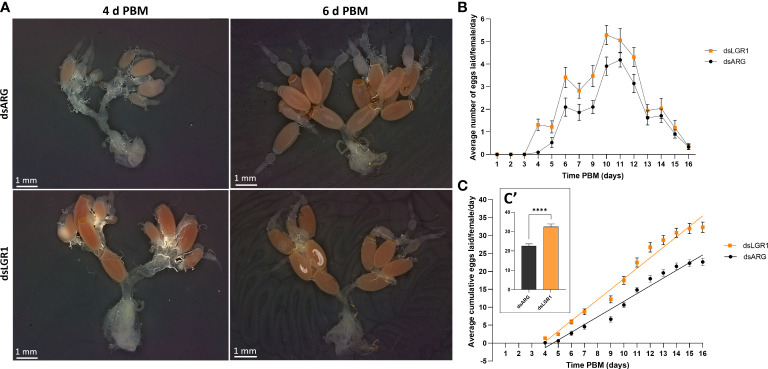
Effects of LGR1 mRNA knockdown on egg laying.**(A)** Representative images showing the reproductive system from dsARG and dsLGR1-injected insects 4 d PBM and 6 d PBM. Note that dsLGR1-injected insects have less eggs in the ovaries at 6 d PBM since they have an accelerated rate of egg laying when compared with dsARG-injected insects. **(B)** The average eggs laid per female each day (error bars indicates ± SEM; n = 17-21). **(C)** The cumulative average of eggs laid per female throughout the 16 d PBM. The significance of differences between linear regression slopes of dsLGR1-injected and dsARG-injected groups was determined using an F-test (P = 0.0001). **(C′)** the cumulative average of eggs laid per female by 16 d PBM. Statistically significant differences were determined by t-test (****p < 0.0001).

**Figure 6:**
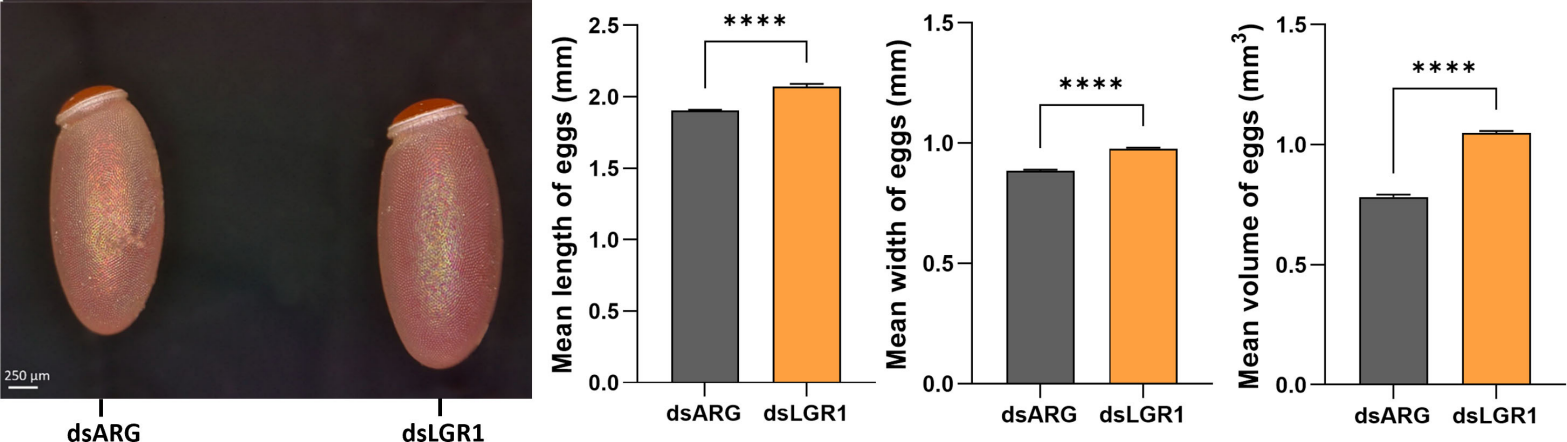
Effects of *LGR1* mRNA knockdown on egg phenotype from recently laid eggs. Representative images on the left panel displaying the eggs 1-2 days post egg-laying from dsLGR1-injected insects and dsARG-injected insects. Graphs on the right panel show the measurements of the length, width, and volume of eggs. Results are shown as mean ± SEM, n = 15 eggs. Statistically significant differences were determined by Student's t-test (****p < 0.0001).

Egg production was evaluated in dsARG-injected and dsLGR1-injected insects by quantifying the duration over which eggs are produced, the number and phenotype of eggs produced, and their hatching rate. *LGR1* knockdown resulted in accelerated egg production, and by 4d PBM there were already chorionated eggs in the ovaries and lateral oviducts ready for oviposition, as compared to the controls (**Figure 5A**). At 6d PBM, both groups were egg laying, but the number of eggs laid by the dsLGR1-injected insects was greater (**Figure 5B**) resulting in the number of eggs remaining in the ovaries being less than in the control insects (**Figure 5A**). The average number of eggs laid by each female per day for dsLGR1-injected insects is greater than the controls from 4d PBM to 12d PBM and similar from 13d PBM to 16d PBM (**Figure 5B**). The slope of the average cumulative number of eggs laid per day per female for dsLGR1-injected insects is significantly higher compared to the slope of the dsARG-injected insects (**Figure 5C**) and the overall cumulative average of eggs laid per female by 16 d PBM is significantly greater than the control (**Figure 5C′**). Interestingly, the length and width of the eggs laid by *LGR1* knockdown insects was significantly greater than control insects, as was the volume of the eggs laid (**Figure 6**).

### 3.5 Influence of LGR1 signaling on the production and release of the yolk protein precursor, vitellogenin

**Figure 7:**
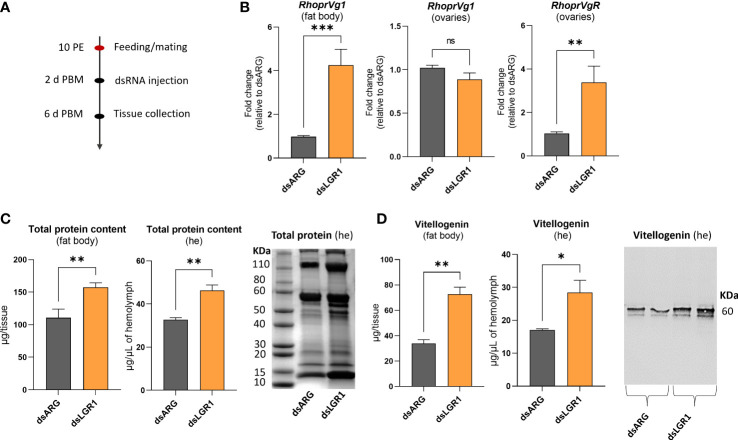
Effects of *LGR1* knockdown on vitellogenesis at 6d post blood meal (PBM). **(A)** Experimental scheme. **(B)** *RhoprVg1* and *RhoprVgR* mRNA expression in the fat body and ovaries of fed adult females at 6 d PBM after dsLGR1 injection. Transcript levels were quantified using RT-qPCR and analyzed by the 2^-ΔΔCt^ method. The y axes represent the fold change in expression relative to control (dsARG, value ~ 1) obtained *via* geometric averaging using *Rp49* and *actin* as reference genes. The results are shown as the mean ± SEM (n = 5-6, where each n represents an individual tissue from 1 insect). **(C)** Protein content in the fat body and hemolymph (he) of dsRNA-injected females at 6 d PBM. The results are shown as the mean ± SEM (n = 5-6, where each n represents the fat body or hemolymph from 1 insect). The SDS-PAGE analysis of hemolymph (1 μL) after downregulation of *LGR1*. Image representative of 3 independent experiments. **(D)** Quantification of vitellogenin in the fat body and hemolymph (he) of dsRNA-injected females measured by ELISA. The results are shown as the mean ± SEM (n = 5-6, where each n represents the fat body or hemolymph from 1 insect). Western blot image showing vitellogenin in the hemolymph of dsRNA-injected females (1 μL of he at a 1:20 dilution). Image representative of 3 independent experiments. Statistically significant differences were determined by student’s t-test. ns, not significant, i.e. p > 0.05; *p < 0.05; **p < 0.01; ***p < 0.001.

To further examine the involvement of LGR1 signaling in egg production, we measured transcript expression of *R. prolixus* vitellogenin (*RhoprVg1*) in the fat body and ovaries, as well as the vitellogenin receptor (*RhoprVgR*) transcript in the ovaries following *LGR1* knockdown (**Figure 7**). *RhoprVg1* transcript levels in the fat body and *RhoprVgR* transcript levels in the ovaries are significantly increased at 6 d PBM in dsLGR1-injected insects (**Figure 7B**). No difference in *RhoprVg* transcript levels was detected in the ovaries after dsLGR1 knockdown. In addition, the total protein content in the fat body and circulating in the hemolymph of dsLGR1-injected insects is greater than in the controls, and these differences are also observed on the Coomassie blue stained gels (**Figure 7C**). The changes in total protein content are due in part to the increase in the main yolk protein precursor (YPP), vitellogenin, in the fat body and hemolymph, as determined by ELISA and Western blot (**Figure 7D**).

### 3.6. Effects of *LGR1* transcript knockdown on hatching of eggs

**Figure 8:**
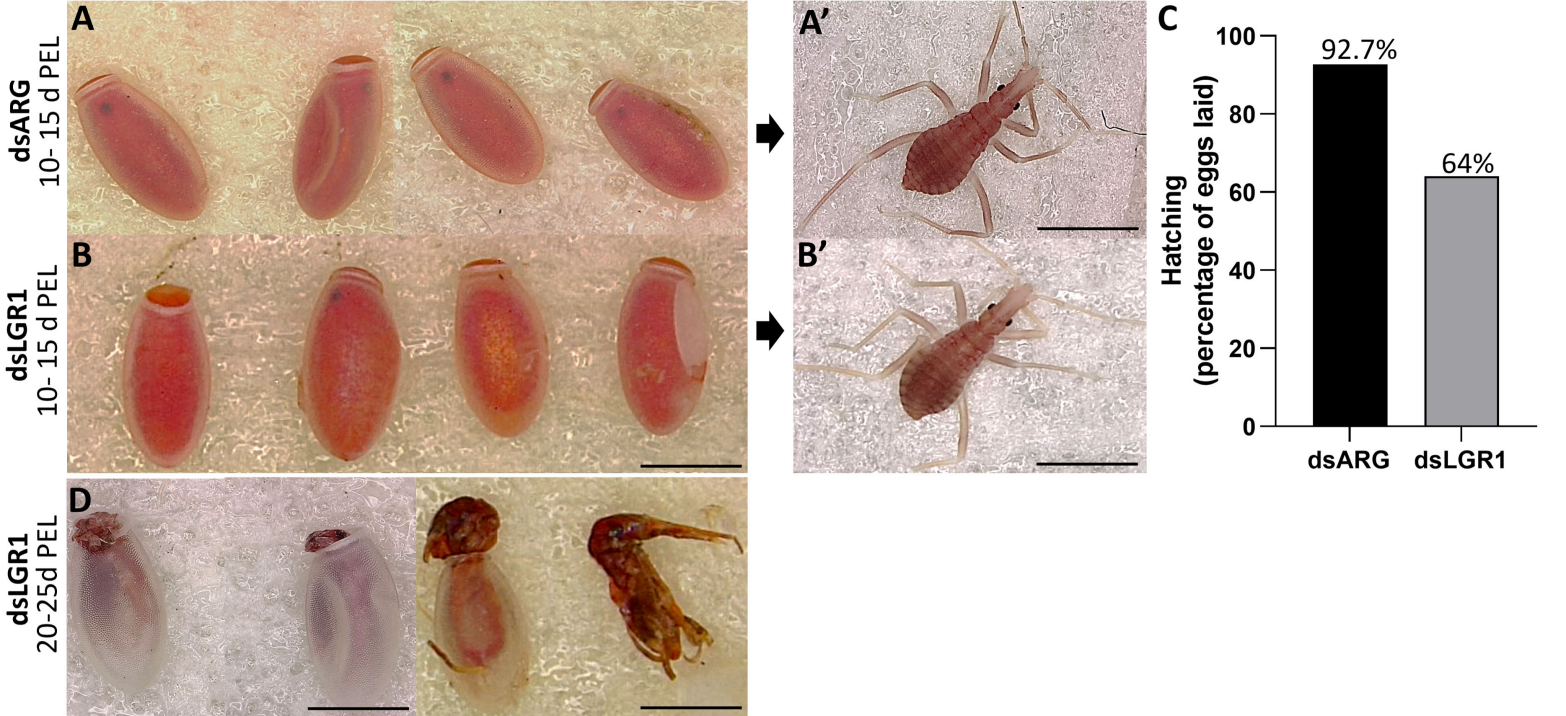
Effect of dsRNA treatment on hatching. **(A)** Egg phenotype at 10-15 days post egg laying (PEL) for dsARG-injected insects, **(A)** shows a 1^st^ instar from a hatched egg. Representative image from n = 10 insects. **(B)** Egg phenotype at 10 - 15 days PEL from dsLGR1-injected insects, **(B)** shows a 1^st^ instar from a hatched egg. Representative image from n = 10 insects. **(C)** Percentage of hatching from eggs laid for dsARG- injected and dsLGR1-injected insects (475 eggs from dsARG treated and 550 from dsLGR1 treated insects). **(D)** Displays unsuccessful hatching at 20-25 days post egg laying for dsLGR1-injected insects. Scale bars: 1 mm for all images.

The effects of LGR1 knockdown on hatching was observed (**Figure 8**). The eggs laid by dsARG-injected and dsLGR1-injected insects looked similar, as did the 1^st^ instars (**Figure 8** A-A’ and B-B’). Interestingly, though, in the dsARG-injected insects, 92.4% of the eggs hatched 10 to 15 days after egg-laying, whereas in dsLGR1-injected insects only 64% of eggs hatched within the same time period (**Figure 8C**). Of the eggs that did not hatch in the LGR1 knockdown insects, some of them attempted to hatch within the next few days but were unsuccessful, with some instars trapped in the egg case and others dying in the process of hatching (**Figure 8D**). It is also worth noting that only 6% of dsLGR1-injected insects, which successful hatched to first instars, survived up to 60 days in the unfed state as opposed to 55% survival for dsARG-injected first instars (data not shown). There was no difference in size between insects that successfully hatched from dsLGR1-injected females when compared to insects hatched from dsARG-injected females.

## 4 Discussion

In *R. prolixus*, egg production is coordinated by neuropeptides and several well-characterized hormonal signaling pathways including juvenile hormones (JHs), ecdysteroids, and insulin-like peptides (ILPs) (see 27). In this study we focus on the role of a new signaling pathway involving the glycoprotein hormone, GPA2/GPB5 and its receptor LGR1, in reproduction, examining the effects of *LGR1* downregulation on egg production in adult females *R. prolixus.*

The distribution of GPB5 in the CNS and reproductive tissues of the adult female *R. prolixus* was observed using immunohistochemistry. A similar distribution pattern of neurons in the CNS of adult *R. prolixus* matches that previously reported for fifth instar *R. prolixus* (14); particularly in relation to neurosecretory cells and their abdominal nerve neurohemal sites, indicative of a neurohormonal role. GPB5-like immunoreactivity is also present in axons within the trunk nerves that project to various regions of the reproductive system, with GPB5-like immunoreactive processes overlying the oviducts, ovaries, spermatheca and bursa, suggesting a possible direct neural control over muscle contraction or some other physiological process. Interestingly, GPB5-like staining is also associated with the fine muscle fibers on the ovarioles, matching a previous report that found *GPB5* transcript in muscle tissues of amphioxus (35).

Another interesting GPB5-like expression pattern is observed in the tropharium, intracellularly in trophocytes, in zones where germ cells undergo mitotic growth (36), possibly suggesting that the GPA2/GPB5 signaling pathway may play a role during the mitotic division of germ cells in the adult *R. prolixus* ovaries. The blebs and processes seen localized on the follicles and the staining within the trophocytes are also indicative of this signaling pathway’s possible involvement in oocyte development or the passage of the transcript into the oocyte *via* the trophic cord. Egg development in the previtellogenic stage starts at the onset of adult female emergence, and following a blood meal, juvenile hormone, ecdysteroids, and insulin-like peptides are released and initiate vitellogenesis and other processes. In *R.* prolixus a blood meal is essential to provide the required nutrients (lipids and proteins) for successful oogenesis, including the production of vitellogenin by the fat body and its accumulation into the oocyte (27). Given that GPB5-like staining is observed in the cytoplasm shared by multiple nurse cells in unfed *R. prolixus*, this suggests GPA2/GPB5 may be involved in physiological processes prior to vitellogenesis. The intercellular compartment of nurse cells has previously been shown to express transcripts for *LGR1* in *A. aegypti* and for the insulin receptor and insulin/ToR signaling in *R. prolixus*, suggestive of local control over egg development (37, 38).

*GPA2*, *GPB5*, and *LGR1* transcripts are found in a variety of tissues, including reproductive tissues of the adult female *R. prolixus*. Interestingly, the highest expression levels of *LGR1* are found in Malpighian tubules, suggesting a possible involvement of this glycoprotein hormone signaling in aspects of water balance and stress tolerance as shown in Al-Dailami etal. (14). The difference in *GPA2* and *GPB5* transcript distribution patterns has been seen in mammals (8, 17). In humans, for example, *GPA2* transcripts are present in the pituitary gland, ovary, testis, kidney, and pancreas, whereas *GPB5* transcripts are mainly restricted to the pituitary gland and the brain (8). Although the high *R. prolixus GPA2* and *GPB5* transcript levels in the CNS supports heterodimerization, the differences in tissue distribution profiles and expression levels could also suggest that these subunits might act as homodimers. Elevated levels of *LGR1* transcripts in the fat body and reproductive tissues of adult female *R. prolixus* PBM further suggests the involvement of this glycoprotein hormone signaling pathway in oocyte development and in regulating the production of nutrients by the fat body for vitellogenesis. In *R. prolixus*, YPPs are primarily synthesized by the fat body during vitellogenesis, and these YPPs, including the main YPP, vitellogenin, are taken up by growing oocytes. Indeed, knocking down *LGR1* results in a significant increase in *RhoprVg1* expression in the fat body and increased *RhoprVgR* expression in the ovaries. This is also evident from the total protein content of dsLGR1-treated insects, which shows an accumulation in the fat body and in the hemolymph, with vitellogenin as a major contributor. The increased *VgR* transcript expression in the ovaries would enable a greater uptake of the hemolymph vitellogenin. Interestingly, the transcript expression of *Vg1* in the ovaries is not increased by dsLGR1 treatment and so is not the major source of accumulated vitellogenin in the oocyte. The egg size in dsLGR1 treated *R. prolixus* is significantly greater than in the controls. This suggests that downregulation of *LGR1* signaling accelerates and increases the amount of YPPs produced by the fat body and taken up by the developing eggs. Furthermore, knockdown of *LGR1*increases the number of eggs produced in a shorter period of time after feeding compared to the control, and in addition to the larger size of eggs, the cumulative number of eggs produced is increased. Downregulating *LGR1* in the adult female *R. prolixus* also leads to a lower hatching rate and unsuccessful hatching in a significant number of eggs. Insects that died during hatching tended to be unable to pass successfully through the operculum, indicating a possible disruption of the normal hatching behavior. Interestingly, although the size of eggs from the dsLGR1-treated and control insects were different, those that successfully hatched in the dsLGR1-treated insects had similar body morphology and body length. In addition, even though 64% of the dsLGR1-treated insects hatched, they had lower survival in the subsequent 60 days, suggesting transgenerational effects from downregulating *LGR1* the adult females.

In accordance with the data described above, similar experiments in the female prawn, *M. rosenbergii*, found that *MrLGR1* transcript knockdown also increase *MrVgR* transcript expression in the ovaries, resulting in larger oocytes, and suggesting to the authors that GPA2/GPB5 acts as a gonad-inhibiting factor in the eyestalk-hepatopancreas-ovary endocrine axis (23). In female *R. prolixus*, dsLGR1 treatment might also alter the onset of oogenesis, which normally starts at approximately 3 days PBM, leading to an accelerated oogenesis and earlier egg laying. GPA2/GPB5 hormone signaling could therefore be involved in controlling egg production by initially inhibiting the synthesis and release of vitellogenin from the fat body and signaling the female reproductive tissues to delay egg production until digestion of the blood meal is completed and nutrients are fully available. Interestingly this signaling pathway has also been shown to be involved in the male reproductive system in *A. aegypti* adult males, with dsLGR1 treated males having a reduced number of spermatozoa with shortened flagella, and a reduced fertility rate (22).

In conclusion, these results, coupled with the earlier data of Al-Dailami etal. (14) suggest that in adult female *R. prolixus*, the GPA2/GPB5 signaling pathway may be activated by a blood meal and act to delay egg production, possibly until nutrients from the blood meal are available for vitellogenesis. Disruption of this pathway by RNAi leads to accelerated oogenesis, an increase in the number of eggs produced and laid, an increase in egg size and a reduction in hatching rate. Furthermore, the first instars hatching from eggs of adult females that had been injected with dsLGR1 had lower survival in the first 60 days.

Targeting the GPA2/GPB5 signaling pathway may well be a fruitful line of research to pursue. RNAi has the potential to curb vector transmission by *R. prolixus*, targeting their reproductive physiology (39). This control method involves the genetic modification of *R. prolixus* gut microbiota expressing dsRNA specific for *R. prolixus* genes, which produces systemic RNAi affecting development and fecundity in a paratransgenic approach, creating sustainable vector control.

## Data availability statement

The original contributions presented in the study are included in the article/**Supplementary Material**. Further inquiries can be directed to the corresponding author.

## Author contributions

ANA-D, IO, and ABL designed the experiments and mapped out the manuscript. ANA-D performed the experiments, analyzed data, wrote the manuscript, and prepared all the figures. IO and ABL contributed to writing and revisions of the manuscript, figures, and data analysis. All authors reviewed and approved the final version of the manuscript.

## Supplementary material

Supplementary Figure 1 Verification of dsRNA efficiency by real time qPCR (n=5). Transcript levels of *LGR1* in fat body and reproductive system of fed insects at 2 days (d), 4 d, and 14 d post injection (PI) are significantly reduced compared to the controls (dsARG-injected insects). Statistically significant differences were determined by Student’s t-test. *p< 0.05, **p< 0.01, ***p< 0.001, ****p< 0.0001.

Supplementary Figure 2 Controls for immunohistochemistry. **(A–F)** Control treatments where GPB5 antibody was preabsorbed with GPB5 antigen. A) Representative image of the mesothoracic ganglionic mass (MTGM) and abdominal nerves of the central nervous system showing an absence of GPB5-like immunoreactivity in neurons, or processes on the abdominal nerves. **(B–C)** Representative images of the the common and lateral oviducts with no GPB5-like immunoreactivity. **(D–E)** Representative images of the tropharium of the ovariole stained with DAPI (blue) with reduced staining intensity for GPB5-like immunoreactivity (green), and (F) no positively stained processes on the bursa. Similar results obtained from 4 preparations. Scale bars: **(A–B)** 50 µm, **(C)** 20 µm, **(D–F)** 50 µm.

## References

[1] Pierce JG Parsons TF. Glycoprotein hormones: Structure and function. Annu Rev Biochem (1981) 50:1. doi: 10.1146/annurev.bi.50.070181.002341 6267989

[2] Kerr JB Sharpe RM. Follicle-stimulating hormone induction of leydig cell maturation. Endocrinol. (1985) 116:6. doi: 10.1210/endo-116-6-2592 3922745

[3] Stockell Hartree A Renwick AG. Molecular structures of glycoprotein hormones and functions of their carbohydrate components. Biochem J (1992) 287:3. doi: 10.1042/bj2870665 PMC1133060 1445230

[4] Dierich A Sairam MR Monaco L Fimia GM Gansmuller A LeMeur M. Impairing follicle-stimulating hormone (FSH) signaling *in vivo*: Targeted disruption of the FSH receptor leads to aberrant gametogenesis and hormonal imbalance. PNAS. (1998) 95:23. doi: 10.1073/pnas.95.23.13612 PMC24867 9811848

[5] Combarnous Y. Molecular basis of the specificity of binding of glycoprotein hormones to their receptors. Endocr. Rev (1992) 13:4. doi: 10.1210/edrv-13-4-670 1281088

[6] Hauser F Søndergaard L Grimmelikhuijzen CJP. Molecular cloning, genomic organization and developmental regulation of a novel receptor from *Drosophila melanogaster* structurally related to gonadotropin-releasing hormone receptors from vertebrates. Biochem Biophys Res Commun (1998) 249:3. doi: 10.1006/bbrc.1998.9230 9731220

[7] Vibede N Hauser F Williamson M Grimmelikhuijzen CJP. Genomic organization of a receptor from sea anemones, structurally and evolutionarily related to glycoprotein hormone receptors from mammals. Biochem Biophys Res Commu. (1998) 252:2. doi: 10.1006/bbrc.1998.9661 9826559

[8] Hsu SY Nakabayashi K Bhalla A. Evolution of glycoprotein hormone subunit genes in bilateral metazoa: Identification of two novel human glycoprotein hormone subunit family genes, GPA2 and GPB5. Mol Endocrinol (2002) 16:7. doi: 10.1210/mend.16.7.0871 12089349

[9] Nakabayashi K Matsumi H Bhalla A Bae J Mosselman S Hsu SY. Thyrostimulin, a heterodimer of two new human glycoprotein hormone subunits, activates the thyroid-stimulating hormone receptor. J Clin Invest. (2002) 109:11. doi: 10.1172/jci0214340 PMC150994 12045258

[10] Dos Santos S Mazan S Venkatesh B Cohen-Tannoudji J Quérat B. Emergence and evolution of the glycoprotein hormone and neurotrophin gene families in vertebrates. BMC Evol Biol (2011) 11:1. doi: 10.1186/1471-2148-11-332 22085792 PMC3280201

[11] Nishi S Hsu SY Zell K Hsueh AJ. Characterization of two fly LGR (leucine-rich repeat-containing, G protein-coupled receptor) proteins homologous to vertebrate glycoprotein hormone receptors: Constitutive activation of wild-type fly LGR1 but not LGR2 in transfected mammalian cells. Endocrinol. (2000) 141:11. doi: 10.1210/endo.141.11.7744 11089539

[12] Sudo S Kuwabara Y Park J-I Hsu SY Hsueh AJ. Heterodimeric fly glycoprotein hormone-α2 (GPA2) and glycoprotein hormone-β5 (GPB5) activate fly leucine-rich repeat-containing G protein-coupled receptor-1 (DLGR1) and stimulation of human thyrotropin receptors by chimeric fly GPA2 and human GPB5. Endocrinol. (2005) 146:8. doi: 10.1210/en.2005-0317 15890769

[13] Paluzzi J-P Vanderveken M O’Donnell MJ. The heterodimeric glycoprotein hormone, GPA2/GPB5, regulates ion transport across the hindgut of the adult mosquito, *Aedes aegypti* . PloS One (2014) 9:1. doi: 10.1371/journal.pone.0086386 PMC3896475 24466069

[14] Al-Dailami AN Leyria J Orchard I Lange AB. Exploring the role of glycoprotein hormone GPA2/GPB5 in the medically important insect, *Rhodnius prolixus* . Peptides (2022) 149. doi: 10.1016/j.peptides.2021.170710 34915093

[15] Okada SL Ellsworth JL Durnam DM Haugen HS Holloway JL Kelley ML. A glycoprotein hormone expressed in corticotrophs exhibits unique binding properties on thyroid-stimulating hormone receptor. Mol Endocrinol (2006) 20:2. doi: 10.1210/me.2005-0270 16210345

[16] Bassett JH van der Spek A Logan JG Gogakos A Bagchi-Chakraborty J Murphy E. Thyrostimulin regulates osteoblastic bone formation during early skeletal development. Endocrinol. (2015) 156:9. doi: 10.1210/en.2014-1943 PMC4541616 26018249

[17] Sun S-C Hsu P-J Wu F-J Li S-H Lu C-H Luo C-W. Thyrostimulin, but not thyroid-stimulating hormone (TSH), acts as a paracrine regulator to activate the TSH receptor in mammalian ovary. J Bio.Chem. (2010) 285:6. doi: 10.1074/jbc.m109.066266 PMC2823517 19955180

[18] Heyland A Plachetzki D Donelly E Gunaratne D Bobkova Y Jacobson J. Distinct expression patterns of glycoprotein hormone subunits in the *Lophotrochozoan aplysia*: Implications for the evolution of neuroendocrine systems in animals. Endocrinol. (2012) 153:11. doi: 10.1210/en.2012-1677 PMC3473217 22977258

[19] Vandersmissen HP Van Hiel MB Van Loy T Vleugels R Vanden Broeck J. Silencing *D. melanogaster* LGR1 impairs transition from larval to pupal stage. Gen Comp Endocrinol (2014) 209:135-47. doi: 10.1016/j.ygcen.2014.08.006 25157788

[20] Rocco DA Paluzzi J-PV. Functional role of the heterodimeric glycoprotein hormone, GPA2/GPB5, and its receptor, LGR1: An invertebrate perspective. Gen Comp Endocrinol (2016) 234:20-7. doi: 10.1016/j.ygcen.2015.12.011 26704853

[21] Sellami A Agricola H-J Veenstra JA. Neuroendocrine cells in *Drosophila melanogaster* producing GPA2/GPB5, a hormone with homology to LH, FSH and TSH. Gen Comp Endocrinol (2011) 170:3. doi: 10.1016/j.ygcen.2010.11.015 21118692

[22] Rocco DA Garcia AS Scudeler EL dos Santos DC Nóbrega RH Paluzzi J-PV. Glycoprotein hormone receptor knockdown leads to reduced reproductive success in male *Aedes aegypti* . Front Physiol (2019) 10:266. doi: 10.3389/fphys.2019.00266 30941056 PMC6433794

[23] Wahl M Levy T Manor R Aflalo ED Sagi A Aizen J. Genes encoding the glycoprotein hormone GPA2/GPB5 and the receptor LGR1 in a female prawn. Front Endocrinol (2022) 13:823818. doi: 10.3389/fendo.2022.823818 PMC8990981 35399936

[24] Bern C Messenger LA Whitman JD Maguire. Chagas disease in the united states: A public health approach. Clin Microbiol Rev (2019) 33:1. doi: 10.1128/cmr.00023-19 PMC6927308 31776135

[25] Lidani KC Andrade FA Bavia L Damasceno FS Beltrame MH Messias-Reason IJ. Chagas disease: From discovery to a worldwide health problem. Front Public Health (2019) 7:166. doi: 10.3389/fpubh.2019.00166 31312626 PMC6614205

[26] Rocco DA Paluzzi J-PV. Expression profiling, downstream signaling, and inter-subunit interactions of GPA2/GPB5 in the adult mosquito *Aedes aegypti* . Front Endocrinol (2020) 11:158. doi: 10.3389/fendo.2020.00158 PMC7137729 32296389

[27] Lange AB Leyria J Orchard I. The hormonal and neural control of egg production in the historically important model insect, *Rhodnius prolixus*: A review, with new insights in this post-genomic era. Gen Comp Endocrinol (2022) 321-322:114030. doi: 10.1016/j.ygcen.2022.114030 35317995

[28] Orchard I Leyria J Al-Dailami A Lange AB. Fluid secretion by malpighian tubules of *Rhodnius prolixus*: Neuroendocrine control with new insights from a transcriptome analysis. Front Endocrinol (2021) 12:722487. doi: 10.3389/fendo.2021.722487 PMC8426621 34512553

[29] Livak KJ Schmittgen TD. Analysis of relative gene expression data using real-time quantitative PCR and the 2–ΔΔCT method. Methods. (2001) 25:4. doi: 10.1006/meth.2001.1262 11846609

[30] Church SH Donoughe S de Medeiros BA Extavour CG. Insect egg size and shape evolve with ecology, not developmental rate. Nature (2019) 571. doi: 10.1101/471946 31270484

[31] Leyria J El-Mawed H Orchard I Lange AB. Regulation of a trehalose-specific facilitated transporter (TRET) by insulin and adipokinetic hormone in *Rhodnius prolixus*, a vector of chagas disease. Front Physiol (2021) 12:624165. doi: 10.3389/fphys.2021.624165 33643069 PMC7902789

[32] Aguirre SA Frede S Rubiolo ER Canavoso LE. Vitellogenesis in the hematophagous *Dipetalogaster maxima* (Hemiptera: Reduviidae), a vector of chagas’ disease. J Insect Physiol (2008) 54:2. doi: 10.1016/j.jinsphys.2007.10.012 18068184

[33] Leyria J Orchard I Lange AB. Impact of JH signaling on reproductive physiology of the classical insect model, *Rhodnius prolixus* . Int J Mol Sci (2022) 23:22. doi: 10.3390/ijms232213832 PMC9692686 36430311

[34] Sedra L Lange AB. The female reproductive system of the kissing bug, *Rhodnius prolixus*: arrangements of muscles, distribution and myoactivity of two endogenous fmrfamide-like peptides. Peptides (2014) 53:140-47. doi: 10.1016/j.peptides.2013.04.003 23598080

[35] Wang P Liu S Yang Q Liu Z Zhang S. Functional characterization of Thyrostimulin in Amphioxus suggests an ancestral origin of the signaling pathway. Endocrinol (2018) 159:10. doi: 10.1210/en.2018-00550 30192937

[36] Nunes-da-Fonseca R Berni M Tobias-Santos V Pane A Araujo HM. *Rhodnius prolixus*: From classical physiology to modern developmental biology. Genesis. (2017) 55:5. doi: 10.1002/dvg.22995 28432816

[37] Rocco DA Kim DH Paluzzi J-PV. Immunohistochemical mapping and transcript expression of the GPA2/GPB5 receptor in tissues of the adult mosquito, *Aedes aegypti* . Cell Tissue Res (2017) 369:2. doi: 10.1007/s00441-017-2610-3 28401307

[38] Leyria J Orchard I Lange AB. The involvement of insulin/tor signaling pathway in reproductive performance of *Rhodnius prolixus* . Insect Biochem Mol Bio. (2021) 130:103526. doi: 10.1016/j.ibmb.2021.103526 33453353

[39] Whitten MM Facey PD Del Sol R Fernández-Martínez LT Evans MC Mitchell JJ. Symbiont-mediated RNA interference in insects. Proc R (2016) 283. doi: 10.1098/rspb.2016.0042 PMC4810840 26911963

