# Supplementary figures and images for: Investigating the role of glycoprotein hormone GPA2/GPB5 signaling in reproduction in adult female Rhodnius prolixus
Source: Front Insect Sci. 2022 Dec 22;2:1096089. doi: 10.3389/finsc.2022.1096089 (PMC10926448; doi:10.3389/finsc.2022.1096089)

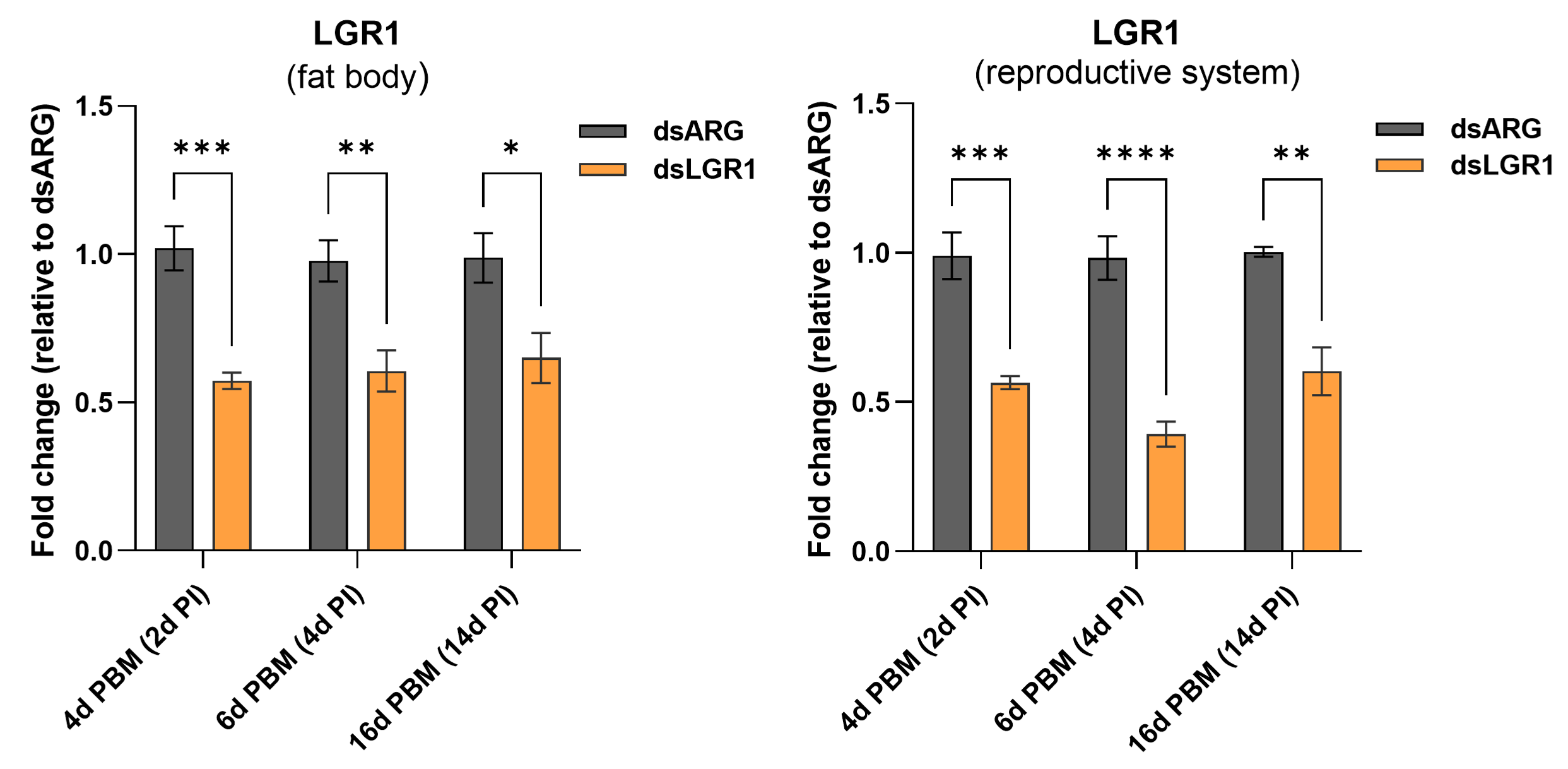

Supplement: Supplementary Figure 1 — Verification of dsRNA efficiency by real time qPCR (n=5). Transcript levels of LGR1 in fat body and reproductive system of fed insects at 2 days (d), 4 d, and 14 d post injection (PI) are significantly reduced compared to the controls (dsARG-injected insects). Statistically significant differences were determined by Student’s t-test. *p< 0.05, **p< 0.01, ***p< 0.001, ****p< 0.0001. [file Image_1.tif]

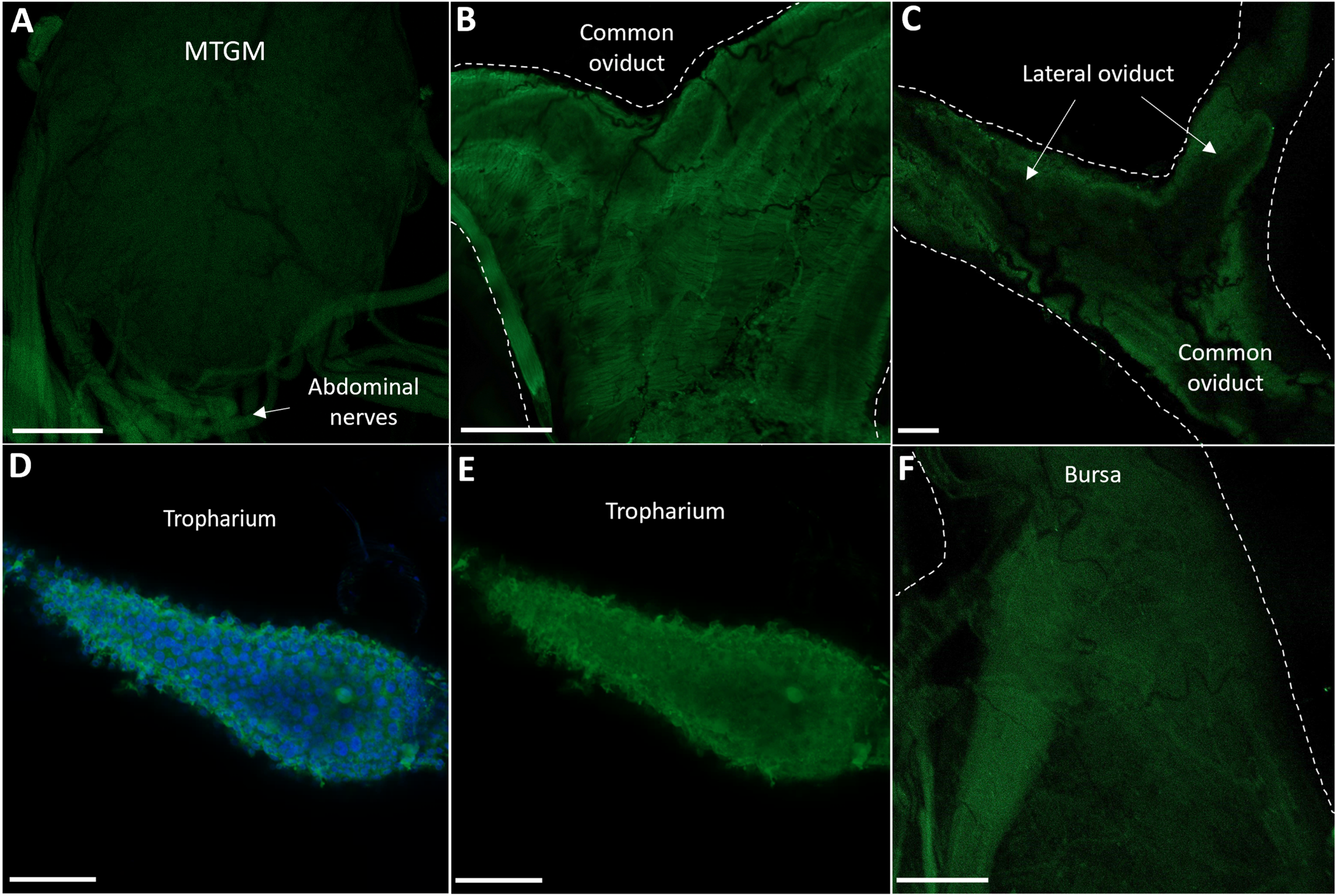

Supplement: Supplementary Figure 2 — Controls for immunohistochemistry. (A–F) Control treatments where GPB5 antibody was preabsorbed with GPB5 antigen. A) Representative image of the mesothoracic ganglionic mass (MTGM) and abdominal nerves of the central nervous system showing an absence of GPB5-like immunoreactivity in neurons, or processes on the abdominal nerves. (B–C) Representative images of the the common and lateral oviducts with no GPB5-like immunoreactivity. (D–E) Representative images of the tropharium of the ovariole stained with DAPI (blue) with reduced staining intensity for GPB5-like immunoreactivity (green), and (F) no positively stained processes on the bursa. Similar results obtained from 4 preparations. Scale bars: (A–B) 50 µm, (C) 20 µm, (D–F) 50 µm. [file Image_2.tif]
