# Supplementary material for: Investigating the role of glycoprotein hormone GPA2/GPB5 signaling in reproduction in adult female Rhodnius prolixus
Source: Front Insect Sci. 2022 Dec 22;2:1096089. doi: 10.3389/finsc.2022.1096089 (PMC10926448; doi:10.3389/finsc.2022.1096089)
Supplement: Supplementary file 3 [file Table_1.docx]

Suppl. Table 1. Gene-specific primers used for qPCR and dsRNA experiments.

| **Oligo Name** | **Oligo Sequence 5’- 3’** |
| --- | --- |
| GPB5 F  GPB5 R | CGGGCACTGAACAGTATGATT  TTGGTACAAGCGTAGCATTCC |
| GPA2 F  GPA2 R | ACACTAACGCTTGTCGAGGA  ACCAATGGAAGTAACCGCCT |
| Vg1 F  Vg1 R  VgR F  VgR R | TTGCTAGTCGCATGAACCTG  TTTAGTGGTGCATCGCTCTG  GTGAAACTCAGGAGAAATTGGC  AGGACACACCATGCGCTATC |
| Rp49 F  Rp49 R | ACCAATGGAAGTAACCGCCT  AGGACACACCATGCGCTATC |
| Actin F  Actin R | AGAGAAAAGATGACGCAGATAATGT  ATATCCCTAACAATTTCACGTTCG |
| LGR1 F  LGR1 R  LGR1(2) F  LGR1(2) R  ARG F  ARG R  LGR1 F_T7  LGR1 R_T7  LGR1 (2) F_T7  LGR1 (2) R_T7 | CCGCCAGATAATGGACCTTGT  TTCGTGAATCATGTGTTCTTCA  ATTATAATGGCGGCCCTACC  TGGGCATATCGCGTACATAA  ATGAGTATTCAACATTTCCGTGTC  AATAGTTTGCGCAACGTTG  **TAATACGACTCACTATAGGGAGA**CCGCCAGATAATGGACCTTGT **TAATACGACTCACTATAGGGAGA**TTCGTGAATCATGTGTTCTTCA  **TAATACGACTCACTATAGGGAGA** ATTATAATGGCGGCCCTACC  **TAATACGACTCACTATAGGGAGA** TGGGCATATCGCGTACATAA |
| ARG F_T7  ARG R_T7 | **TAATACGACTCACTATAGGGAGA**ATGAGTATTCAACATTTCCGTGTC  **TAATACGACTCACTATAGGGAGA**AATAGTTTGCGCAACGTTG |

* TAATACGACTCACTATAGGGAGA = T7 RNA polymerase promoter region
